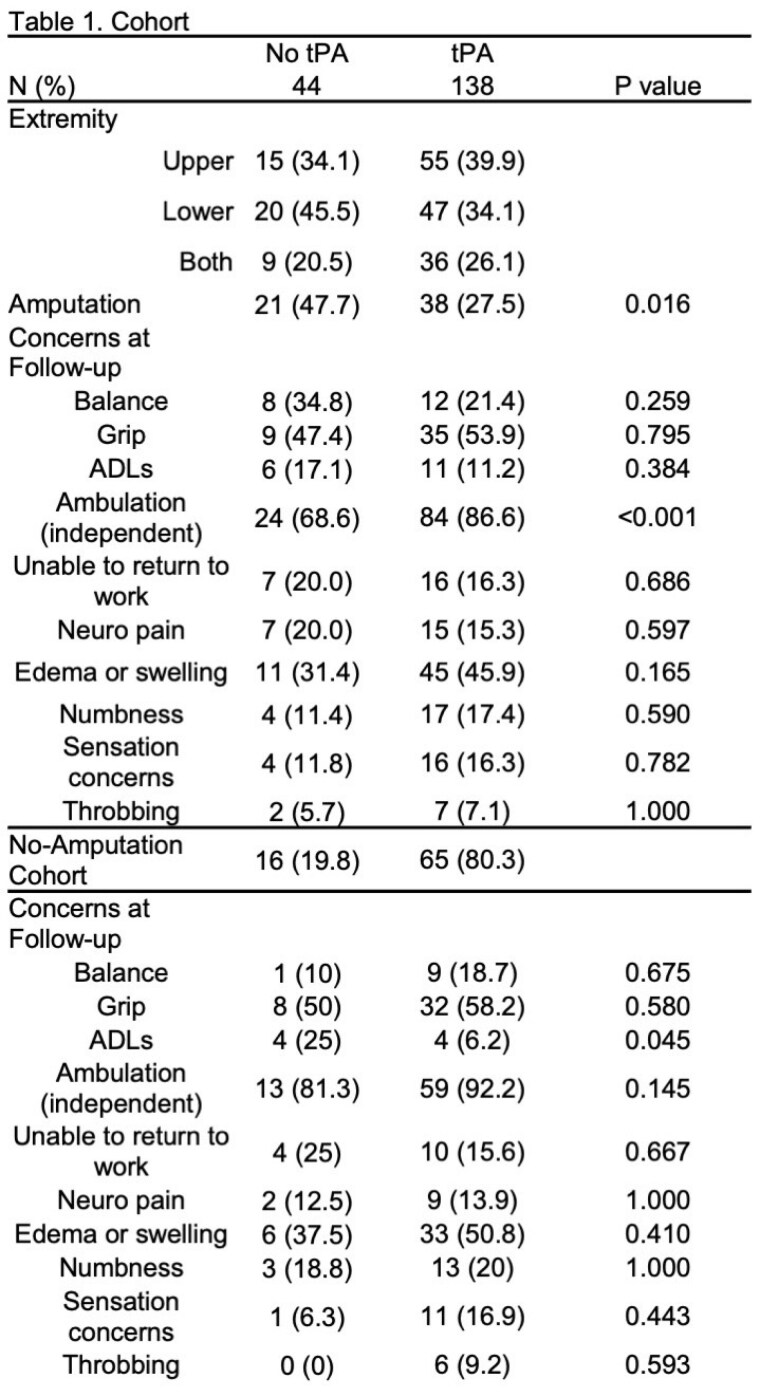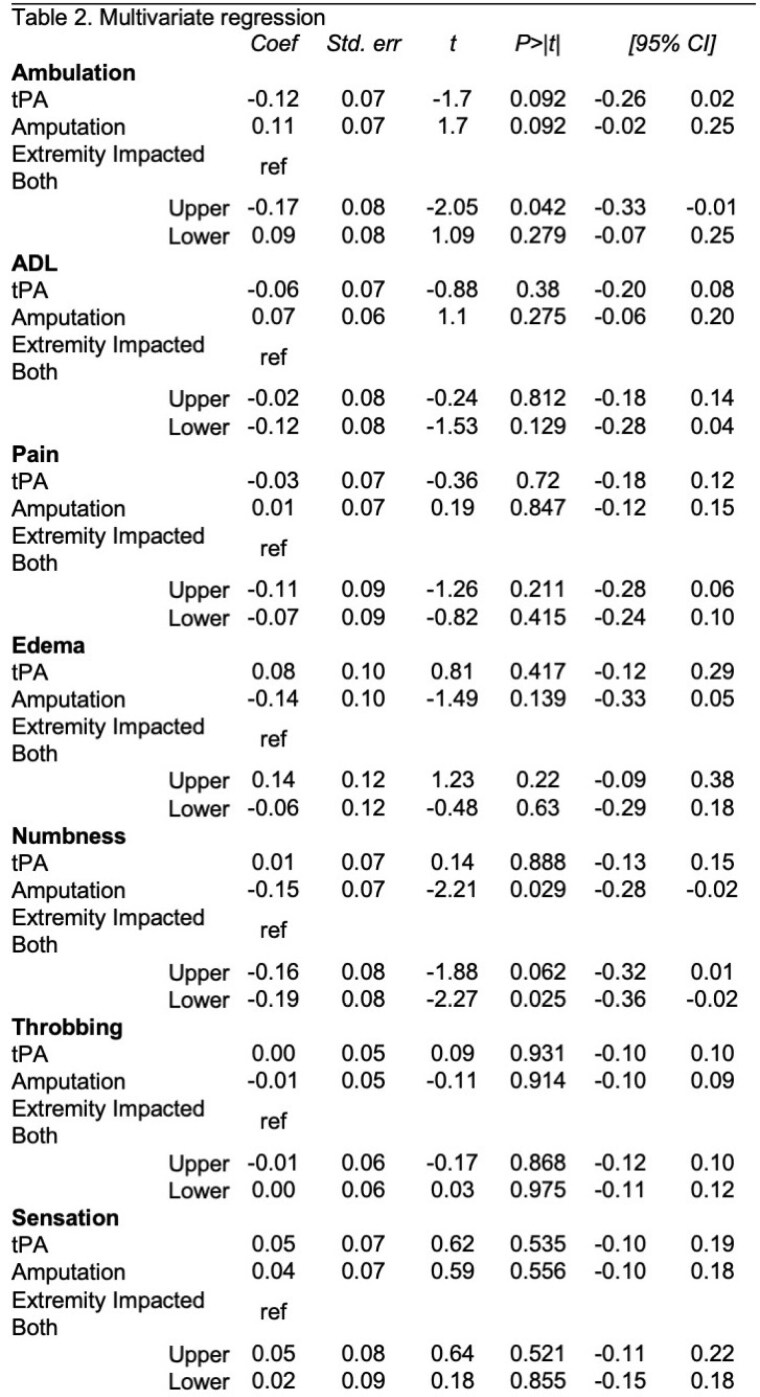# 578 Impact of Thrombolytic Treatment on Functional Outcomes in Severe Frostbite Patients

**DOI:** 10.1093/jbcr/iraf019.207

**Published:** 2025-04-01

**Authors:** Lexy Kindt, Charly Vang, Emily Colonna, Rediat Tilahun, Sarah Zemek, Jana Almendinger, Lisa Nelson, Patricia White, Kyle Schmitz, Derek Lumbard, Rachel Nygaard

**Affiliations:** Hennepin County Medical Center; Hennepin Health Care Research Institute; Hennepin Healthcare; Hennepin Healthcare Research Institute; Hennepin Healthcare; Hennepin County Medical Center; Hennepin Healthcare; Hennepin County Medical Center; Hennepin Healthcare; Hennepin Healthcare; Hennepin Healthcare

## Abstract

**Introduction:**

Thrombolytics (tPA) improve limb and digit salvage following severe frostbite injury, however disability following tPA therapy is largely undescribed. Our aim was to evaluate functional status, pain, and disability in a large cohort of severe frostbite patients treated with and without tPA, hypothesizing no significant differences between the two groups.

**Methods:**

Longitudinal cohort study of severe frostbite injured patients over the winters of 2013-20. Therapy notes were reviewed at discharge and follow-up. Outcomes were categorized including issues with balance, issues with grip or range of motion, requiring assistance or modifications for activities of daily living (ADLs), ambulation status (independent, assist-device, non-ambulatory), and return to work. Presence of pain, edema, numbness, throbbing, and pain medication requirements were also noted. Fisher’s exact test and multivariate regression evaluated independent predictors (tPA, amputation, and injury location) on 7 outcome variables measured.

**Results:**

The cohort included 182 patients, 75.8% received tPA. Of those with lower extremity frostbite, balance issues were documented in 64.3% and 69.9% of patients (no tpa vs tPA, p=0.641). Issues with grip, range of motion, or stiffness were reported in 60.9% and 78.9% of upper extremity injuries (no tPA vs tPA, p=0.103). Over a third of patients required assistance with ADLs (32.6% no tPA vs 46.7% tPA, p=0.115). Return to work status as same prior to injury were equal between the two treatment groups. Reports of neurologic pain, edema, and numbness were all higher in the tPA treated group, while throbbing was higher in the non-tPA group, but none reached significance. Significantly more non-tPA treated patients required amputation (Table 1). Of those with at least 1 clinic follow-up (n=133), 98 were treated with tPA. Higher proportions tPA patients were independently ambulating at follow-up. No significant differences in reported pain, edema, swelling, sensitivity, or throbbing between the treatment groups were observed at follow-up. When examining those treated with or without tPA that did not require amputation, the results mirrored the cohort. Significantly more non-tPA treated patients without amputation needed assistances with ADLs compared to those treated with tPA (Table 1). Multivariate regression revealed no significant outcome differences between tPA-treated and non-tPA groups, though predictive power was low (Table 2).

**Conclusions:**

This is the first long-term evaluation of functional outcomes of tPA treated severe frostbite patients. Thrombolytics is a valuable intervention in preventing amputation following severe frostbite injury and functional recovery of salvaged limbs and digits warrant additional study.

**Applicability of Research to Practice:**

Tailored rehabilitation interventions to mitigate disability and improve ambulation outcomes should be prioritized for severe frostbite injured patients.

**Funding for the Study:**

N/A